# Do Radiographic Assessments of Periodontal Bone Loss Improve with Deep Learning Methods for Enhanced Image Resolution?

**DOI:** 10.3390/s21062013

**Published:** 2021-03-12

**Authors:** Maira Moran, Marcelo Faria, Gilson Giraldi, Luciana Bastos, Aura Conci

**Affiliations:** 1 Policlínica Piquet Carneiro, Universidade do Estado do Rio de Janeiro, Rio de Janeiro 20950-003, Brazil; mfaria@uerj.br (M.F.); odonto@uerj.br (L.B.); 2 Instituto de Computação, Universidade Federal Fluminense, Niterói 24210-310, Brazil; 3 Faculdade de Odontologia, Universidade Federal do Rio de Janeiro, Rio de Janeiro 21941-617, Brazil; 4 Laboratório Nacional de Computação Científica, Petrópolis 25651-076, Brazil; gilson@lncc.br

**Keywords:** periodontal imaging, resolution improvement, visual quality, neural networks, deep-learning

## Abstract

Resolution plays an essential role in oral imaging for periodontal disease assessment. Nevertheless, due to limitations in acquisition tools, a considerable number of oral examinations have low resolution, making the evaluation of this kind of lesion difficult. Recently, the use of deep-learning methods for image resolution improvement has seen an increase in the literature. In this work, we performed two studies to evaluate the effects of using different resolution improvement methods (nearest, bilinear, bicubic, Lanczos, SRCNN, and SRGAN). In the first one, specialized dentists visually analyzed the quality of images treated with these techniques. In the second study, we used those methods as different pre-processing steps for inputs of convolutional neural network (CNN) classifiers (Inception and ResNet) and evaluated whether this process leads to better results. The deep-learning methods lead to a substantial improvement in the visual quality of images but do not necessarily promote better classifier performance.

## 1. Introduction

The visual quality of imaging examinations is a major factor impacting the diagnosis process for several oral diseases. This quality is essential, since accurate identification of anatomical substructures, pathologies, and functional features depends on it. Diagnostic errors affect treatment planning and are a huge risk to the health of patients. Even though the advent of digital systems promoted better quality for examinations, many issues can result in low-quality images. Previous works assessed the visual quality characteristics observed by experts when they evaluated images [1,2]. Along with their overall appearance, experts considered features such as radio density, edge definition, image contrast, and resolution—this last one being mainly related to the sensor’s capacity.

Regarding periodontal imaging, the use of periapical radiographs can be considered an important tool in this area, helping in the diagnosis, treatment, and prognosis of periodontal diseases [1,2,3,4,5,6]. Moreover, periapical radiographs are considered the standard for evaluating periodontal bone loss (PBL), since the facilitates the identification of bone defects [3,4,5,6,7,8]. This kind of examination usually covers not only the entire set of teeth, from their roots to their crowns, but also their adjacent bones. For this type of examination, low resolution is also an issue, since the corresponding acquisition tools have limitations.

In the scope of radiographic image acquisition, high spatial resolutions demand high ionizing radiation doses. Based on the popularity of radiographic examinations, many studies have emphasized the harmful effects of X-rays [9]. However, due to the importance of imaging in oral disease assessments, these side effects should not prevent the use of radiographs for diagnosis. Nevertheless, efforts should be made to maintain the minimum possible radiation dose with the minimum image quality loss [9]. Radiation is absorbed by patients subjected to any radiographic technique, so the risks (and benefits) to which the patient will be exposed during the exams are widely established. Additionally, the quantification of this dose rate and the quality of the radiation used are defined by specific legislation around the world, which regulates the use of ionizing radiation, thereby aiming to obtain the best image with the lowest possible dose for the diagnosis. One alternative is to apply computer-based resolution improvements to low-resolution images previously obtained. Baskan et al. [10] demonstrated that specific computer-based resolution improvements allow some radiation dose reductions while resulting in similar image quality. In clinical practice, experts mainly use zoom tools provided by commercial software (based on interpolation methods) as an alternative for increasing the resolution of low-resolution examinations.

The use of improved resolution, along with the available zoom tools, facilitates identifying several periodontal diseases [11]. The study presented by Morais et al. [11] suggests that PBL diagnosis efficiency and resolution improvement, promoted by zoom tools, present a positive correlation.

Nevertheless, the interpolation methods on which the mentioned zoom tools are based tend to add artifacts and undesired blur and aliasing effects so that there is an upper limit for the improvements that can be achieved by them. Previous studies [12] demonstrated that using such tools to achieve high magnification factors may actually have a negative impact, reducing the visual definition of edges, and thereby impacting the assessments of anatomical and lesion structures, and consequently the diagnosis. This negative effect on image quality also affects the users’ perceptions of image fidelity [12].

In the study presented by Morais et al. [11], zooming was used to achieve magnifications of 2 ×, 3 ×, 4 ×, and 8 ×, then the accuracies in the detection of simulated periodontal bone defects were compared. The bone defects used in that work covered the coronal part of the interproximal area between the first and second premolars. The images achieved by the 8 × magnification were discarded due to their low quality, which limited the PBL assessment. An expert analyzed the treated images considering the presence of periodontal bone defects. The accuracy and the area under the receiver operating characteristic curve (AUC_ROC_) pointed out that the considered magnifications had similar performances in detecting periodontal bone defects. However, the AUC_ROC_ values had slight decreases as the magnification increased, which suggests that this type of resolution improvement has no significant positive impact on PBL diagnosis. This could be due to the quality limitations previously mentioned (artifact addition and blur and aliasing effects).

The use of different resolution improvement methods can also affect automatic processes. The work presented by Alvares [13] analyzed the impacts of using different interpolation methods on the results of the segmentation of skin lesions performed by convolutional neural networks (CNNs) [14]. Moreover, it demonstrated that there are significant differences between the interpolation methods in this application, which suggests that using appropriate resolution improvements in the input images can positively impact the performances of deep-learning methods. Previous works also assessed the impacts of low-quality input images on the performances of deep-learning methods [15,16]. Dodge and Karam [15] evaluated how different aspects, such as blur, noise, contrast, and compression, can impact the performances of VGG-CNN-S [17], GoogleNet [18], and VGGNet [19] classifier models. The results obtained in the aforementioned studies demonstrated that the tested networks are sensitive to blur effects, which, according to the authors, can be related to the interference in the images’ textures used by CNNs to identify patterns, and consequently, objects. Additionally, Koziarski and Cyganek [16] evaluated how using low-resolution input images affect the AlexNet [20], VGGNet, and ResNet networks in an image recognition task (Large Scale Visual Recognition Challenge 2012). The results presented in that study show that using resolution improvements leads to an increase in CNNs’ classification accuracy compared with using low-resolution images.

Recently, deep-learning-based methods have been proposed to achieve high-resolution images with better quality [21,22,23,24,25,26]. Moreover, some of these methods, specifically the super-resolution convolutional neural network (SRCNN) and the super-resolution generative adversarial network (SRGAN), have also been employed to periapical images in previous work [21].

This work’s main objective was to perform a complete evaluation, considering both qualitative and quantitative analysis, of how different super-resolution algorithms (nearest, bilinear, bicubic, Lanczos, SRCNN, and SRGAN) used on periapical images can impact the assessment of periodontal bone loss. We observed the perceptual quality of the images provided by such methods (essential for the human assessment) and these methods’ effects in pattern recognition automatic algorithms. This was innovative work, since no previous works evaluated the effects of deep-learning resolution improvement methods on assessing oral diseases. For this analysis, we performed two studies.

In the first one, a set of five periapical radiographs were treated with the six super-resolution methods. The resultant images were visually evaluated considering their subjective qualities. Their scores were compared using the visual grading characteristics (VGC) curve [27].

In the second study, we extracted a set of regions of interest (ROI) in periapical images covering the interproximal area and treated them with the considered methods. Then, we trained convolutional neural networks with these treated images to classify whether the regions presented any interproximal bone loss or not. In that way, we found our whether using the images treated with deep-learning led to better performance in the classification task.

## 2. Radiographic Identification of Periodontal Bone Loss

Radiographs act as a complement to clinical examinations in the assessment of PBL and periodontal diseases in general [1,2,28]. In most cases, radiographs reveal features that are difficult to assess clinically, such as advanced periodontal lesions [28]. Nevertheless, radiographs also present some limitations. For example, bone destruction tends to appear less severe than it actually is, resulting in undetected mild destructive lesions, since they do not change the tissue density, and consequently the radiodensity, enough to be detectable in an exam [28]. Early bone changes can be identified in radiographs as subtle, mild erosions in the interproximal alveolar bone crest. These erosions tend to appear as very slights changes, but this does not mean that the disease process is recent, since the loss must occur for 6 to 8 months before radiographic evidence becomes visible [28]. When these early bone changes progress, they evolve into more severe bone loss, which can be identified as an increase in radiolucency due to a decrease in tissue density.

**Figure 1:**
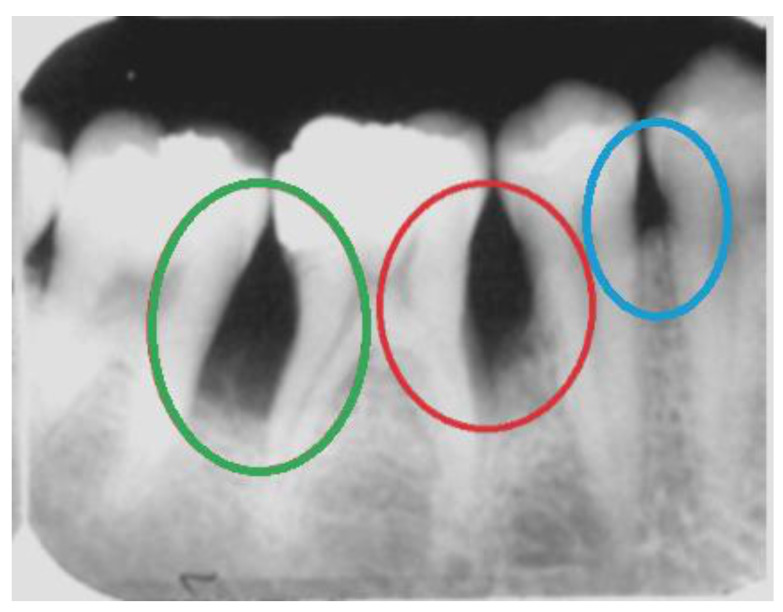
Examples of bone loss in a real periapical radiograph: an interproximal crater in green, vertical bone loss in red, and a horizontal bone defect in blue.

In this work, we focus on horizontal bone loss, vertical bone defects, and interdental craters. These patterns of bone loss may be visible radiographically. In general, interproximal bone loss can be radiographically and clinically observed as an increase in the distance from the enamel–cement junction to the alveolar crest. Horizontal bone loss consists of a horizontal loss in the alveolar bone’s height; i.e., the tissue destruction is symmetrical. Radiographically, vertical bone loss can be identified as a deformity in the alveolus extending apically along the root of the affected tooth from the alveolar crest. When it happens in an interproximal region between two teeth, it can be seen as an uneven lesion, more accentuated on one side. The interproximal crater consists of a lesion that radiographically can be observed as a two-walled, trough-like depression. This loss has a band-like or irregular appearance in the interdental region between adjacent teeth [28]. Figure 1 shows examples of these three types of bone loss.

### Convolutional Neural Networks for PBL Identification

The last few years have seen an intensification of machine learning methods being used to support diagnosis in several medical conditions. Moreover, previous works demonstrated the feasibility of using neural networks to identify or classify periodontal diseases in radiographs [29,30,31,32,33,34]. Convolutional neural networks (CNNs) were applied for alveolar bone loss identification and measurement [30], and identification and severity assessment of premolars and molars compromised [31]. Recent works also used CNNs for the classification of periapical lesions, considering their extent [32,33]. Additionally, CNNs were used to detect apical lesions on panoramic dental radiographs [34]. The seven-layer network presented by Ekert et al. [34] achieved a sensitivity value of 0.65, a specificity value of 0.87, a negative predictive value of 0.93, and a positive predictive value of 0.49. CNNs also demonstrated good performance in the detection of PBL on panoramic dental radiographs. A network presented by Krois et al. [8], composed of seven layers, achieved accuracy, sensitivity, and specificity of 0.81 for this problem.

More recently, Moran et al. [29] evaluated two widely used CNN architectures (Inception and ResNet) to classify regions in periapical examinations according to the presence of periodontal bone destruction. The Inception model presented the best results, which were impressive, even considering the small and unbalanced dataset used. The final accuracy, precision, recall, specificity, and negative predictive values were 0.817, 0.762, 0.923, 0.711, and 0.902, respectively. Such results suggest the feasibility of using the CNN model as a clinical decision support tool to assess periodontal bone destruction in periapical exams.

## 3. Deep-Learning Resolution Improvement Methods

As previously mentioned, most commercial tools use interpolation methods for resolution improvement. Nevertheless, in the last few years, there was an expansion of deep-learning methods for this task. As pointed out by Yang et al. [35], super-resolution (spatial resolution improvement problem) solutions can be categorized based on the tasks they focus on, i.e., the specific classes of images they focus on. In this work, we focused on medical imaging, but in order to select the solution to be included in our evaluation, we considered the algorithms’ performances in benchmarks established in the literature.

Deep-learning based super-resolution methods have been widely used in medical imaging applications [21,22,23,24,26,36,37]. Zhang and An [23] proposed a deep-learning solution formed by two convolutional layers preceded by a prefixed bicubic interpolation. The transfer learning technique was also considered. In that work, the authors applied the proposed method to different types of medical images, such as knee magnetic resonance images (MRI), mammography, and angiography. Shi et al. [36] proposed a residual learning-based algorithm for MRI. The method proposed by Zeng et al. [22] also focuses on magnetic resonance images. It is a convolutional neural network that operates two types of super-resolution reconstructions (single and multi-contrast) at the same time. Park et al. [26] presented a super-resolution solution for computed tomography images. It consists of a deep-learning convolutional neural network, based mainly in the U-Net architecture. Zhao et al. [37] proposed SMORE, a deep-learning solution for the visualization improvement of brain lesions in fluid-attenuated inversion recovery images. Resolution improvement methods based on deep learning have also been applied to oral radiographs. Hatvani et al. [24] proposed a super-resolution method for enhancing dental cone-beam computerized tomography. That method is based on tensor-factorization and promotes a two-times magnification increase. Concerning deep-learning methods for resolution improvement, two algorithms have achieved impressive results in several applications, including in medical imaging: the super-resolution convolutional neural network (SRCNN) [38] and the super-resolution generative adversarial network (SRGAN) [39]. These two solutions consist of state-of-the-art methods that achieved the best results for super-resolution in the literature for benchmark datasets.

**Figure 2:**
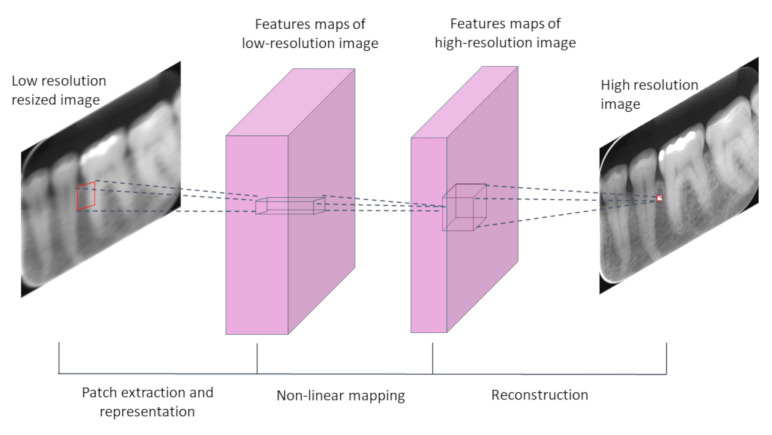
Scheme of the super-resolution convolutional neural network’s (SRCNN) main structure.

The SRCNN was initially proposed for Dong et al. [38] in 2016, and became the state-of-the-art method for super-resolution (considering a 2× magnification factor) for the BSD200 benchmark datasets [40], and due to this outstanding performance, it was included in our evaluation. The SRCNN (Figure 2) requires pre-processing of the inputs before the network handles them, which involves the application of bicubic interpolation to obtain an initial image of the desired resolution. Then, the deep neural network processes the pre-processed images. The network operation is divided into three main steps: 1—patch extraction and representation; 2—nonlinear mapping; 3—reconstruction.

SRCNNs have been used in several resolution improvement tasks. Umehara, Ota, and Ishida [25] proposed a scheme for resolution improvement in chest CT images based on the SRCNN. The method proposed by Qiu et al. [41] is an SRCNN-based reconstruction solution for knee MRIs. It is formed by three SRCNN hidden layers and a sub-pixel convolution layer.

**Figure 3:**
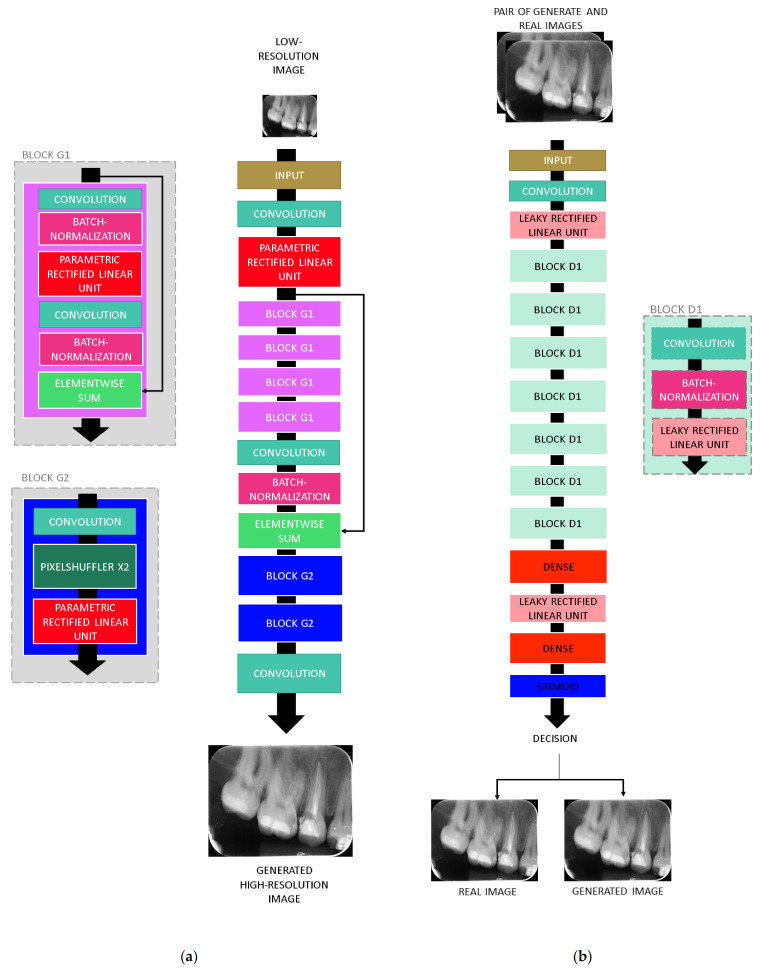
SRGAN architecture: (**a**) generator network and (**b**) discriminator network.

Another popular deep-learning method for resolution improvement is the SRGAN [39]. This solution was proposed in 2017 by Ledig et al. [39] and became the state-of-the-art for super-resolution using the BSD100 and PIRM datasets [40,42], overcoming all other super-resolutions previously mentioned. The current state-of-the-art in this problem is a variation of SRGAN. SRGAN also demonstrated high performance in a wide range of applications [21,43,44,45]. Therefore, this solution was also selected for this analysis. The SRGAN follows the main structure of general generative adversarial networks: it is composed of a generative model G and a differentiable discriminator D (Figure 3). The generator network G is trained as a feed-forward CNN parametrized by θ_G_, where θ_G_ corresponds to the weights and biases of an L-layer, obtained by optimizing a super-resolution-specific loss [39]. In the training process, G is trained to create images that simulate real images and in that way mislead D, which is trained to distinguish between real images and the images generated by G [39]. SRGANs have also been applied to medical imaging. Liu et al. [45] presented an SRGAN to obtain high-resolution brain MRI data. Recently, Moran et al. [21] evaluated the use of an SRGAN for obtaining high-resolution periapical radiographs, considering the transfer learning technique.

Although SRCNN and SRGAN (including their variations) are currently state-of-the-art methods for super-resolution, other algorithms have also shown impressive results. The KK method [46] has also presented good performance when working on the super-resolution problem. The following steps define it: initial rescaling using bicubic interpolation, and high-frequency detail recovery using local patch-based regression. For this last step, the band frequency components are extracted by the Laplacian operation.

The sparse coding (SC) method proposed by Yang et al. [47] consists of defining a sparse representation for each image patch of the low-resolution input to generate the high-resolution output. For that, two dictionaries D_h_ and D_l_ are trained, for low and high-resolution image patches, respectively. The main idea is to obtain the same sparse representations for low and high-resolution versions of the same patch using D_l_ and D_h_. Then, this translation method, composed of the two dictionaries, can be used to obtain high-resolution images from the sparse representations of low-resolution image patches. Using this approach, with sparse representations of patches instead of the actual patches, reduces the method’s computational cost significantly. The anchored neighborhood regression (ANR) method [48] is also based on the sparse representations, generalizing them by allowing the approximation of low-resolution input patches using a linear combination of their nearest neighbors. For that, a neighboring embedding should be defined, considering that the patches’ representations lie on low-dimensional nonlinear manifolds with locally similar geometry.

## 4. Materials and Methods

In order to assess how using deep-learning resolution improvement methods impact the visual quality of periapical images, and consequently, the identification of PBL, we performed two different studies. The Research Ethics Committee approved the studies presented here (CAAE, registered at the Brazilian Ministry of Health, 20703019.8.3001.5259). The periapical radiographs used were acquired in the Policlíınica Piquet Carneiro of Rio de Janeiro State University, using the long cone paralleling technique for minimal distortion. For image acquisition, the Sirona Heliodent Plus device (70 kVp, 7 mA, Kavo Brasil Focus) was used. The exposure time was 0.25 to 0.64 s. In addition to that, the image acquisition used the EXPRESS™ Origo imaging plate system (Intraoral imaging plate system. https://www.kavo.com/dental-xray-machines-diagnostics/intraoral-x-ray, archived on 19 February 2021) by KaVo Dental (Biberach an der Riss, Germany). For both acquisition and storage, the Express digital system was used. The digital image format was the grayscale JPEG.

### 4.1. Study 1—Qualitative Analysis of Image Quality

In the first study, we aimed to evaluate the perceptual quality of the periapical radiographs treated with different methods. The main idea of Study 1 was to assess the order of the approaches used to increase the spatial resolution of dental images according to their quality, promoting an easier assessment of PBL. For that, five periapical radiographs were treated with each of the considered approaches (nearest, bilinear, bicubic, Lanczos, SRCNN, and SRGAN—these last two being deep-learning-based methods obtained by Moran et al. [21]). In total, 30 treated images were considered. Then, we asked observers to evaluate the quality of the treated images considering aspects that impacted their visual analysis of PBL, such as edge definition artifacts, blur, and aliasing. The observers qualified each of the treated images by assigning scores based on the mean opinion score (MOS) metric [49], a perceptual quality metric that considers a scale ranging from 1 to 4. On that scale, 1—denotes poor quality, 2—reasonable quality, 3—good quality, and 4—very high quality. This evaluation was performed asynchronously using an online form.

Concerning the observers included in this study, they can be separated into two groups: experts and lay observers. The expert group was formed by experienced dentists, two of whom were dentists specialized in oral radiography (experts 1 and 2), and two were dentists specialized in endodontics (experts 4 and 5). The lay group was formed by 17 participants who were not dentists or radiologists and can be considered lay in PBL or oral radiography assessments. Eight of them presented previous contact with concepts related to medical images (radiographs and/or other medical images). Additionally, twelve of them had contact with concepts related to image processing techniques. The main idea for including laypeople in the study was to observe whether the quality trends denoted by lay observers would be in agreement with the ones denoted by experts.

### 4.2. Study 2—Evaluation of the Impacts of Pre-Processing on Deep-Learning Based Classification

In the last few years, the use of computational algorithms as assistive tools in the diagnosis of several oral diseases has increased substantially. The applications in this scope cover, among other tasks, the classification of oral images according the presence or absence of a certain lesion. In that way, Study 2 aimed to compare the considered super resolution methods as pre-processing steps for deep neural networks, considering the task of classification of regions of interest in periapical radiographs according to the presence of PBL. The main idea of this study was to evaluate the pre-processing’s impacts on the classification performance of such networks.

The process to obtain these regions of interest, which were classified by the CNNs, was defined by Moran et al. [29], and includes the following steps: pre-processing of the periapical examinations using histogram equalization, manual extraction of regions of interest (interproximal areas between two teeth, limited at the top by the enamel–cement junction and at the bottom by the alveolar crests), and labeling of the regions of interest by experts (experienced dentists—one of them a specialist in oral radiology; no differences existed between their annotations) considering the presence or absence of interproximal PBL.

After obtaining the images of the regions of interest, they were split into three sets: training, validation, and test sets. For the test set, we obtained 52 images of each class (with and without PBL), resulting in 104 regions. The remaining images were subjected to two different data augmentation processes in order to increase the dataset’s size and at the same time reduce the differences in the numbers of samples for the classes. For the PBL class, the data augmentation consisted of horizontal flips. For the healthy class, it consisted of horizontal and vertical flips. Consequently, for the training and validation sets, we obtained 1278 images of regions with PBL and 1344 images of healthy regions. The training–validation ratio was 80:20.

The classification networks considered in this study were ResNet [50] and Inception [18]. The input images for ResNet models must be 224 × 224, as defined in [50]. The input resolution for Inception is 299 × 299 [18]. Nevertheless, the images of the regions of interest, obtained by the previous steps, presented spatial resolution lower than that, so rescaling was demanded in order to allow these images to be used as inputs to the classifiers. We resized all the images to the same resolutions (224 × 224 for ResNet inputs and 299 × 299 for Inception inputs) in order to prepare the data to be processed by the CNN classifiers. For that, the images of the regions of interest (of all training, validation, and test sets) were treated with each one the considered resolution improvement methods.

**Figure 4:**
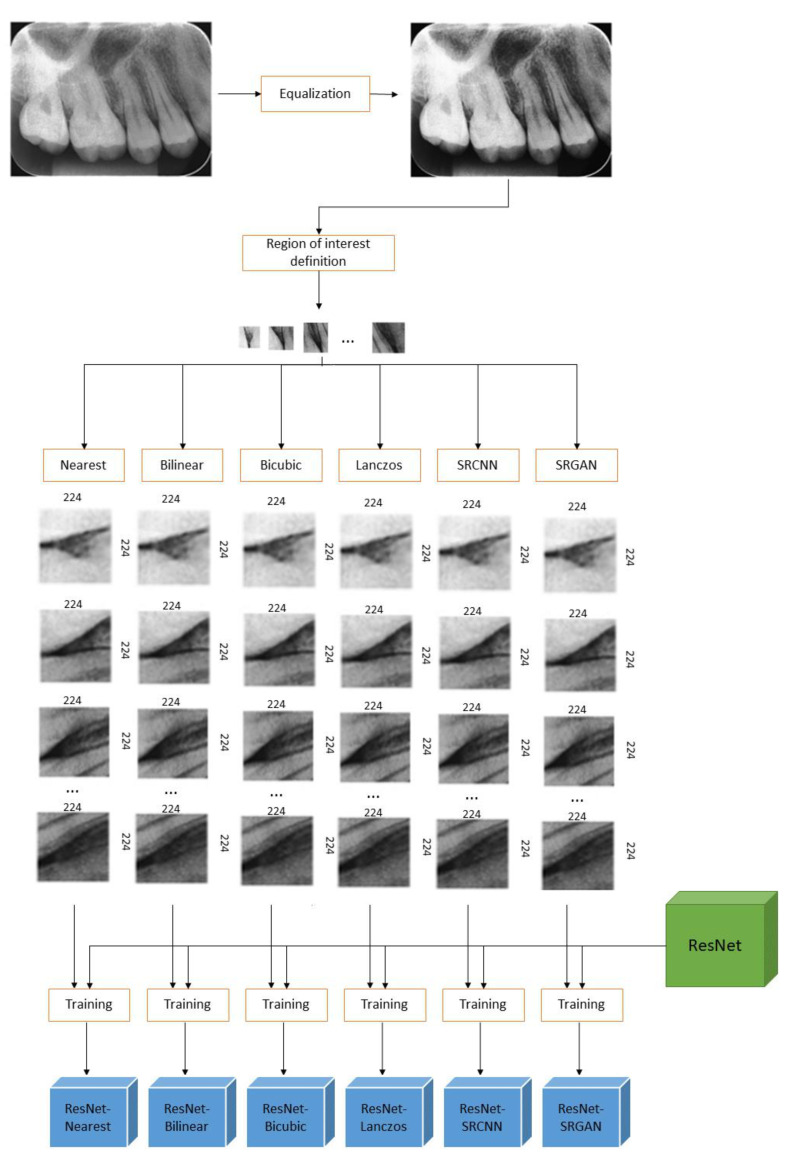
Scheme of Study 2′s methods for ResNet models.

The images obtained for each method were used to train different models, resulting in twelve different models. Six of them were ResNet models, ResNet_Nearest_, ResNet_Bilinear_, ResNet_Bicubic_, ResNet_Lanczos_, ResNet_SRCNN_, and ResNet_SRGAN_, which correspond to the ResNet models trained exclusively with the images treated with the nearest, bilinear, bicubic, Lanczos, SRCNN, and SRGAN methods, respectively. Similarly, the other six models were the Inception models trained exclusively with these same data: Inception_Nearest_, Inception_Bilinear_, Inception_Bicubic_, Inception_Lanczos_, Inception_SRCNN_, and Inception_SRGAN_. Figure 4 shows the whole process for the ResNet models, from the original periapical images to the final trained models to be compared. By analyzing the performances of such classification networks, one can infer which super-resolution method is the best pre-processing rescaling step for these types of classifiers in the defined task.

The classifiers’ training processes were performed using the backpropagation algorithm [51] and included 180 epochs. Transfer learning has been demonstrated to improve the performance of deep-learning methods in classification tasks. In that way, we applied it in the training of our classifiers. All models were initialized using weights obtained by a fine-tuning process considering the Imagenet dataset [52] to achieve better initial weight values, and eliminate the impact of random weight initialization, which could possibly interfere with a classifier’s performance. The training and testing processes were executed in a desktop machine with the following configuration: Intel^®^ Xeon^®^ CPU 2.30 GHz processor by Intel (Mountain View, USA), Tesla P100-PCIE-16GB GPU processor by Nvidia (Santa Clara, USA), 13 GB RAM.

## 5. Results

### 5.1. Study 1

Table 1 shows the general and observers’ MOS based on the answers for: “Evaluate the quality of the radiograph, considering the general quality of the image, visibility of the anatomical structures, definition of the limits between the structures and the presence of artifacts as blur and aliasing.”

To compare the resolution improvement methods, we used the visual grading characteristics (VGC) curve [27], based on the MOS values. As described by Bath [27], the VGC is an evaluation of an image’s subjective characteristics, in which the observer assigns scores to them using a multi-step rating scale (in this case, MOS) in order to state opinions about defined quality criteria. Moreover, it compares the cumulative distributions of the scores of two different methods, providing a general comparison of them concerning a quality criterion. Given a set of images treated with different methods and their respective scores assigned by the observers, it is possible to define a probability distribution of the images from each method. For instance, considering that expert 1 assigned the score 1 to three images treated with the bicubic method (see Table 1, line 4, column 4), the probability of observer 1 assigning score 1 to an image treated with the bicubic method is 3/5 = 0.6 (i.e., 60%), since he classified three images as “poor quality” among the five bilinear images. Similarly, the probability of this same observer (expert 1) assigning “reasonable quality” (score 2) to an image treated with this same method (bicubic) is 2/5 = 0.4, i.e., 40%. In this way, the probability distribution of the bicubic method for observer 1 can be defined as $P_{O_{1}}\left(x\right)$, where $x\;\in \left\{4,3,2,1\right\}$ corresponding to the MOS scores.

$$P_{O_{1}}\left(x\right)=\{\begin{array}{ll} 0.0 & if\;x=4\\ 0.0 & if\;x=3\\ 0.4 & if\;x=2\\ 0.6 & if\;x=1 \end{array}$$

Consequently, the cumulative distribution referent to the bicubic method for observer 1 is defined by function $C_{O_{1}}\left(x\right)$, where x corresponds to the MOS scores:

$$C_{O_{1}}\left(x\right)=\left\{\begin{array}{ll} 0.0 & if\;x=4\\ 0.0 & if\;x=3\\ 0.4 & if\;x=2\\ 1.0 & if\;x=1 \end{array}\right.$$

Using this same idea, we can define the probability distribution of any method M_A_ method for observer n as $P_{M_{A}}^{O_{n}}$, by using the cumulative distribution referent to the M_A_ method for observer n, as $C_{M_{A}}^{O_{n}}$.

By definition, the VGC curve that describes the comparison between two methods M_A_ and M_B_, concerning the grades given by an observer $O_{n}$, is composed of the points whose coordinates are defined by the cumulative distributions $C_{M_{A}}^{O_{n}}$ and $C_{M_{B}}^{O_{n}}$, and can be described as a function $VGC_{M_{A}M_{B}}^{O_{n}}\left(x\right)\;=\;\left(i,j\right)\in {\mathbb{R}}^{2},$ where $i\;=\;C_{M_{A}}^{O_{n}}\left(x\right)$, $j\;=\;C_{M_{B}}^{O_{n}}\left(x\right)$, and $x\;\in \left\{4,3,2,1\right\}$, corresponding to the MOS scores. The higher the area under the VGC curve, the better M_B_ is compared to M_A_.

In this work, we considered fifteen M_A_–M_B_ pairs: nearest–bilinear, nearest–bicubic, nearest–Lanczos, nearest–SRCNN, nearest–SRGAN, bilinear–bicubic, bilinear–Lanczos, bilinear–SRCNN, bilinear–SRGAN, bicubic–Lanczos, bicubic–SRCNN, bicubic–SRGAN, Lanczos–SRCNN, Lanczos–SRGAN, and SRCNN–SRGAN. For each M_A_–M_B_ pair, a total of eight VGC curves were obtained: one for each expert, one considering the scores assigned by all five experts, one considering the scores assigned by all lay participants, and one considering all participants (experts and lay).

**Figure 5:**
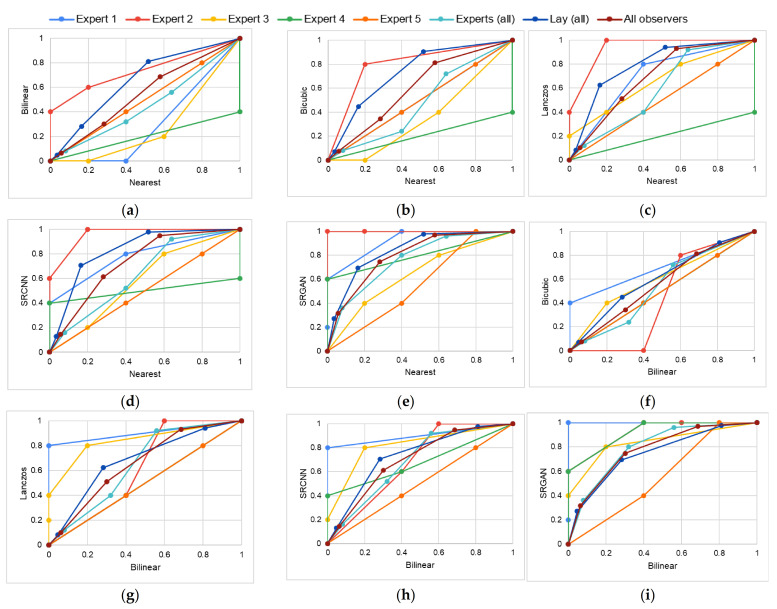

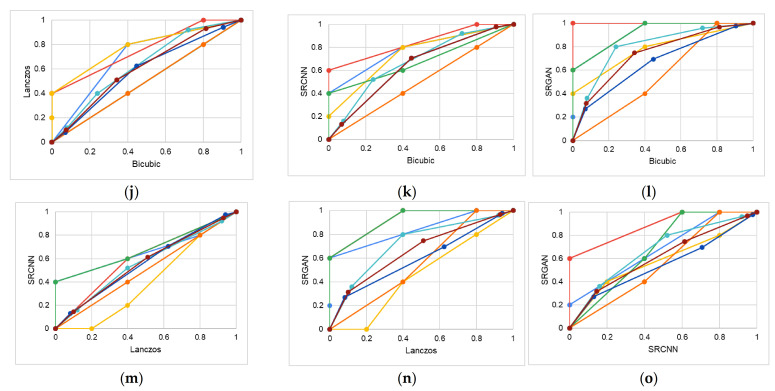
VGC curves obtained for each M_A_–M_B_ pair: (**a**) nearest–bilinear, (**b**) nearest–bicubic, (**c**) nearest–Lanczos, (**d**) nearest–SRCNN, (**e**) nearest–SRGAN, (**f**) bilinear–bicubic, (**g**) bilinear–Lanczos, (**h**) bilinear–SRCNN, (**i**) bilinear–SRGAN, (**j**) bicubic–Lanczos, (**k**) bicubic–SRCNN, (**l**) bicubic–SRGAN, (**m**) Lanczos–SRCNN, (**n**) Lanczos–SRGAN, and (**o**) SRCNN–SRGAN. General VGC curves in brown, VGC curves for all experts in light blue, curves for lay participants in dark blue, expert 1 in medium blue, expert 2 in red, expert 3 in yellow, expert 4 in green, and expert 5 in orange.

The VGC curves obtained for each M_A_–M_B_ pair are shown in Figure 5. They are represented by the plots (a)–(o), where: (a) nearest–bilinear, (b) nearest–bicubic, (c) nearest–Lanczos, (d) nearest–SRCNN, (e) nearest–SRGAN, (f) bilinear–bicubic, (g) bilinear–Lanczos, (h) bilinear–SRCNN, (i) bilinear–SRGAN, (j) bicubic–Lanczos, (k) bicubic–SRCNN, (l) bicubic–SRGAN, (m) Lanczos–SRCNN, (n) Lanczos–SRGAN, and (o) SRCNN–SRGAN. Each color in these plots represents one observer or group: general VGC curves in maroon, VGC curves for all experts in light blue, curves for lay participants in dark blue, expert 1 in medium blue, expert 2 in red, expert 3 in yellow, expert 4 in green, and expert 5 in orange.

Table 2 shows the AUC values of the general VGC curves (obtained considering the scores given by all expert observers and lay participants), the experts VGC curves (obtained considering the scores given by all expert observers), and the lay participants’ VGC curves (obtained considering the scores given by all lay participants). These results denote the superiority of SRGAN.

To evaluate the differences between the M_A_ and M_B_ methods, we applied the non-parametric Wilcoxon paired test [53]. This test is mainly applied to analyze data that present unknown distributions. We considered a 99% confidence interval, and the alternative hypothesis H_A_ was “MOS scores of M_B_ are higher than the values of M_A_.” For that, we considered all scores given by all observers. The Wilcoxon p-value achieved for the nearest–bilinear pair was 0.001—i.e., the p-value considering the H_A_ “bilinear MOS values are higher than nearest MOS values” was < 0.01, so it proved H_A_. The p-values for nearest–bicubic, nearest–Lanczos, nearest–SRCNN, nearest–SRGAN, bilinear–bicubic, bilinear–Lanczos, bilinear–SRCNN, bilinear–SRGAN, bicubic–Lanczos, bicubic–SRCNN, bicubic–SRGAN, Lanczos–SRCNN, Lanczos–SRGAN, and SRCNN–SRGAN were 8.191 × 10^−9^, 1.751 × 10^−11^, 5.175 × 10^−14^, 7.996 × 10^−15^, 2.695 × 10^−5^, 2.406 × 10^−9^, 9.398 × 10^−13^, 2.485 × 10^−12^, 3.795 × 10^−5^, 1.209 × 10^−7^, 2.634 × 10^−9^, 0.007, 1.825 × 10^−6^, and 0.002, respectively, which also prove the hypotheses.

In addition to the subjective evaluation provided by the MOS scores, we also included the following measures: peak signal-to-noise ratio (PSNR), mean square error (MSE), and structural similarity index (SSIM). For that, we downscaled high-resolution images from a 720 × 720 spatial resolution to a 128 × 128 spatial resolution and then applied each one of the considered methods to obtain new images with a 720 × 720 resolution. Then, the similarity of the original high-resolution images and the images obtained by each method was assessed using such measures. Table 3 shows the MSE, PSNR, and SSIM values for the images treated with each super-resolution method, where it is possible to observe that SRGAN provides high-resolution images very close to the original ones, because it presents the lower MSE, the greater PSNR, and the SSIM closest to 1.

### 5.2. Study 2

The confusion matrices for all the ResNet and Inception models are presented in Table 4 and Table 5.

Table 6 and Table 7 show the values achieved for the other metrics considered, which were sensitivity (recall), specificity, precision (positive predictive value, PPV), and negative predictive value (NPV) [54]. In this example, such measures are based on:

- True negatives (*TN*)—regions correctly classified as healthy;
- True positives (*TP*)—regions correctly classified as regions with bone loss;
- False negatives (*FN*)—regions with bone loss incorrectly classified as healthy;
- False positives (*FP*)—healthy regions incorrectly classified as regions with bone loss.

In that way, the mentioned measures are defined as: sensitivity = $\frac{TP}{FN+TP}$; specificity = $\frac{TN}{FP+TN}$; precision = $\frac{TP}{FP+TP}$; negative predictive value = $\frac{TN}{FN+TN}$.

**Figure 6:**
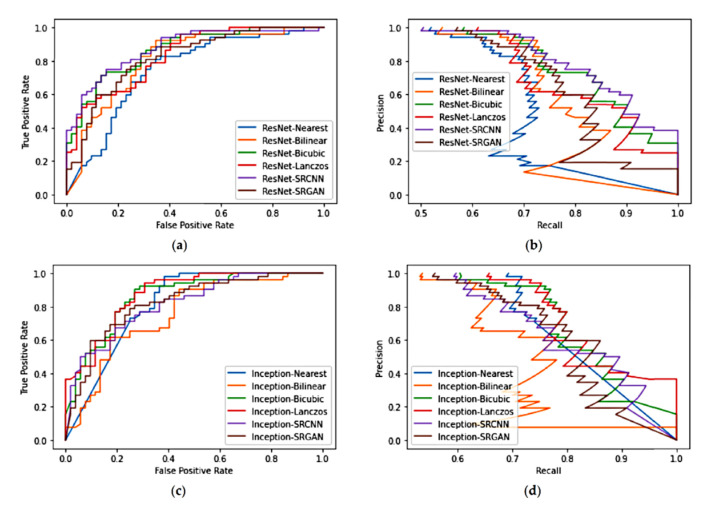
Curves obtained for the considered models. ResNetNearest, ResNetBilinear, ResNetBicubic, ResNetLanczos, ResNetSRCNN, and ResNetSRGAN: (**a**) ROC curves and (**b**) PR curves. InceptionNearest, InceptionBilinear, InceptionBicubic, InceptionLanczos, InceptionSRCNN, and InceptionSRGAN: (**c**) ROC curves and (**d**) PR curves.

Table 8 shows the proportion of correctly classified examples (test accuracy) for each method. Figure 6 shows the receiver operating characteristic (ROC) and precision-recall (PR) curves for each model. Figure 6a shows the ROC curves for ResNetNearest, ResNetBilinear, ResNetBicubic, ResNetLanczos, ResNetSRCNN, and ResNetSRGAN; and Figure 6b shows their PR curves. Similarly, Figure 6c shows the ROC curves for InceptionNearest, InceptionBilinear, InceptionBicubic, InceptionLanczos, InceptionSRCNN, and InceptionSRGAN; and Figure 6d shows their PR curves.

## 6. General Discussion

Relative to Study 1, for expert 3, Lanczos’s quality was very high and very close to SRGAN, which is also denoted by the corresponding VGC curve and its AUC. For expert 4, the nearest method had better results, despite the intense aliasing effect. For expert 5, all interpolation methods and SRCNN had very similar and reasonable performance, except the SRGAN method, which was superior. Except for expert 2, the experts considered the nearest method equal to or better than the bilinear method. This suggests that the nearest aliasing effect tends to be more supported than the bilinear blur effect. For experts 1 and 2, the results’ quality progressively improved for the bilinear, bicubic, Lanczos, SRCNN, and SRGAN methods, as expected. This superiority was also proved by the p-values achieved for the Wilcoxon tests performed. This trend was also observed in a general way, considering the scores for all observers. On the one hand, experts considered SRGAN as the best method and found the effects of bilinear, bicubic, and nearest methods relatively similar. Some of the experts even gave the same scores for those three methods, which can also be seen in the VGC curves that are repeated and overlap for some pairs. Note that the results for experts 4 and 5 differ substantially from the results of other experts, especially for bilinear and bicubic methods. This difference can be mostly related to the fact that they are specialized in endodontics, not in oral radiology, so their perception of the visual quality regarding PBL assessments can differ. Experts 4 and 5 may have had some tolerance for image flaws that were not tolerated by the dentists specialized in oral radiology. Additionally, due to their extensive experience in endodontics and PBL assessment, they may have had some ability to detect PBL even in blurred images.

On the other hand, the two deep-learning methods had very similar performances for laypeople, with SRGAN being slightly better. For the interpolation methods, the improvements had the progression expected in terms of quality, following the order: nearest, bilinear, bicubic, and Lanczos.

**Figure 7:**
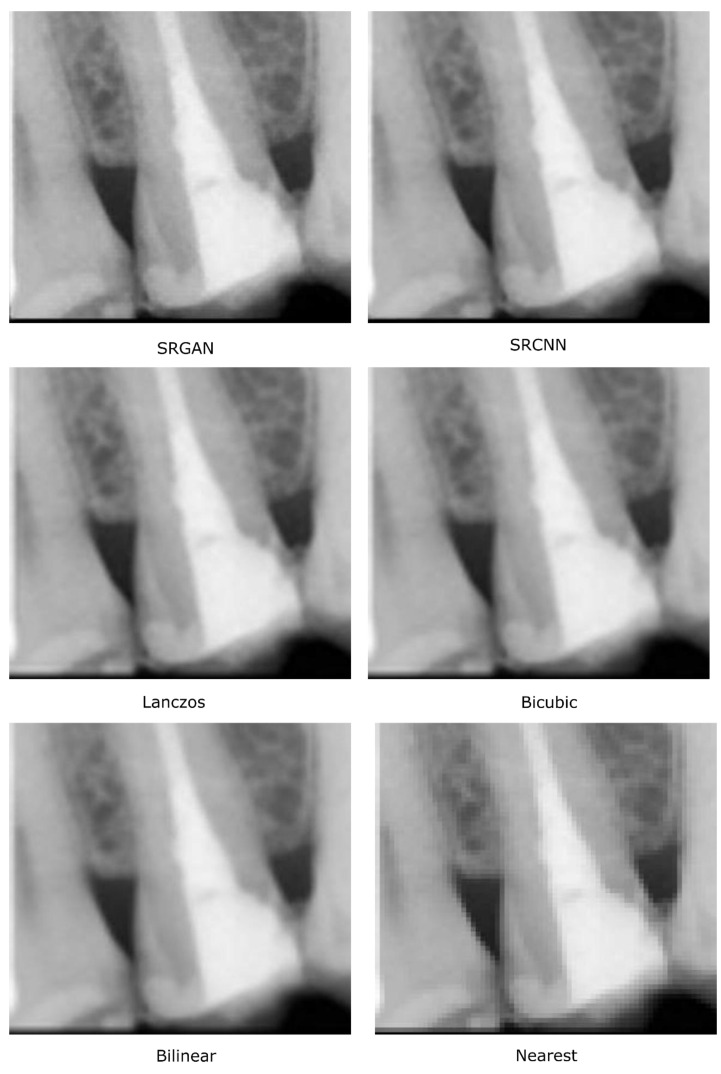
Details (of two interproximal areas) in images obtained by the considered methods.

These visual quality differences are exemplified in Figure 7, in which it is possible to see how the deep-learning methods increase the edges’ definition in the interproximal area.

It is important to emphasize that MOS consists of a qualitative evaluation, so it is a subjective metric that is highly observer dependent. Even while considering that the number of experts in this study can be considered low, observing the general experts’ curves, based on all experts’ scores, minimizes these observer-dependent factors and provides an overview of the perceptual quality. Additionally, the additional analysis performed, including the analysis of lay observers, involved a large number of participants and provided more robust metrics, evidencing more general trends about the quality of the different methods.

Regarding Study 2, at first sight, the classifiers’ performances tend to appear similar in a general way, as denoted by the ROC curves (Figure 6) and the overall accuracy, except for Inception_Bilinear_ and Inception_Bicubic_, which had higher accuracy compared with the other methods. However, in contrast with the results of Study 1, the results of Study 2 for the ResNet models suggest that the use of SRGAN may actually have a bad influence on the classification. The best overall accuracy was obtained by the SRCNN (Table 8). For the ResNet models, Lanczos had a worse accuracy than the bilinear and bicubic interpolation methods. Nevertheless, the bicubic interpolation led to more balanced results, in the way that its performance for both classes was similar (Table 4). For the SRGAN, the accuracy for the healthy class was substantially higher than with other methods, which is reflected by the high precision and specificity values (Table 6). Nevertheless, its low accuracy for the PBL class resulted in many false negatives, which is also denoted by the recall and NPV values. On the other hand, the SRCNN presented the best accuracy for the PBL class, which led to the high recall and NPV values, but its high number of false positives led to low precision and specificity values. Similar phenomena happened to the nearest, bilinear, bicubic, and Lanczos methods. In that way, the ResNet_Nearest_, ResNet_Bilinear_, ResNet_Lanczos_, ResNet_SRCNN_, and ResNet_SRGAN_ models seemed to present a biased trend to the PBL or healthy classes. This might have been caused by the trend that these methods add certain artifacts to this kind of image—blur for bilinear, Lanczos, and SRCNN, and aliasing for nearest and SRGAN [9].

For the Inception models, the use of the deep-learning methods led to worse performance compared with the bicubic method, considering all evaluated metrics (Table 7), which suggests that the mentioned artifacts badly impact the patterns used by models of this architecture. Concerning the interpolation methods, the performance varied largely according to the class considered, denoting a high bias for such Inception models (Table 5 and Table 7).

The results of Study 2 also suggest that advanced CNNs can handle blurred images during the training process in such a way that the pattern recognition is not so drastically affected by this kind of artifact. Moreover, the methods used as pre-processing steps for CNNs should consider factors that impact the pattern recognition algorithms instead of factors that impact human visual perception, since deep learning algorithms perform classification in a different way to how human experts do. Additionally, the development of deep learning algorithms that directly handle low-resolution inputs can be beneficial.

Even considering that in Study 2 we focused on a classification task, using a pre-processing step that improves the spatial resolution of input images is interesting for a wide range of automatic applications. For instance, object detection and segmentation are tasks that could employ such pre-processing. Specifically for segmentation, edges and details definition are essential. One example of an application is segmentation band detections of anatomical structures in CT (or CTA) scans from aortic dissections [55]. In such an application, the enhancement possibly provided by the super-resolution methods may provide more details about the aortic wall and layers, especially in the primary entry tear area, which is demanded in that context.

Concerning the results of both studies, there is evidence that using the deep-learning methods (especially SRGAN) improves the perceptible visual quality of the images in aspects related to the PBL identification, such as contrast and edge definition. On the other hand, their application as a pre-processing step for CNN classifiers did not substantially improve the overall performance. However, it may help to identify more precisely each of the classes, depending on the method used and the classifier considered.

## 7. Conclusions

In this work, we evaluated how using resolution improvement methods influences the assessment of periodontal bone loss. For that, we proposed two different studies, focusing on human and computer-based analysis of PBL, respectively. The results of Study 1 (MOS scores and VGC curves) demonstrated that both deep-learning methods, especially SRGAN, generate high-resolution images with high visual quality in aspects that influence PBL assessment, promoting easier diagnosis. The interpolation methods’ performances varied hugely, but the expected trend was observed in the general evaluation (considering all participants). On the other hand, the deep-learning methods did not substantially improve CNN classifiers’ performances, suggesting that they may add some sort of artifacts that influence the texture patterns that discriminate sample groups along with the CNNs’ operation. We highlight that one of the main limitations of this work was the low number of dentists participating in Study 1.

In future works, we aim to extend this analysis to evaluating the impacts of the deep-learning resolution improvement methods on other computer-based tasks, such as segmentation and object detection.

## Figures and Tables

**Table 1:** Observers’ ratings and respective mean opinion scores (MOS) for the visual grading characteristics (VGC) analysis.

**Expert 1 (specialist in oral radiology)**						
Visual quality perception (MOS score)	Number of cases					
	Nearest	Bilinear	Bicubic	Lanczos	SRCNN	SRGAN
Poor (1)	3	5	3	1	1	0
Reasonable (2)	2	0	2	4	2	2
Good (3)	0	0	0	0	2	2
Very high (4)	0	0	0	0	0	1
**Expert 2 (specialist in oral radiology)**						
Visual quality perception (MOS score)	Number of cases					
	Nearest	Bilinear	Bicubic	Lanczos	SRCNN	SRGAN
Poor (1)	4	2	1	0	0	0
Reasonable (2)	1	1	4	3	2	0
Good (3)	0	2	0	2	3	2
Very high (4)	0	0	0	0	0	3
**Expert 3 (experienced dentist)**						
Visual quality perception (MOS score)	Number of cases					
	Nearest	Bilinear	Bicubic	Lanczos	SRCNN	SRGAN
Poor (1)	2	4	3	2	1	1
Reasonable (2)	2	1	2	2	3	2
Good (3)	1	0	0	1	1	2
Very high (4)	0	0	0	1	0	0
**Expert 4 (specialist in endodontics)**						
Visual quality perception (MOS score)	Number of cases					
	Nearest	Bilinear	Bicubic	Lanczos	SRCNN	SRGAN
Poor (1)	0	0	0	0	0	0
Reasonable (2)	0	3	3	3	2	0
Good (3)	5	2	2	2	1	2
Very high (4)	0	0	0	0	2	3
**Expert 5 (specialist in oral endodontics)**						
Visual quality perception (MOS score)	Number of cases					
	Nearest	Bilinear	Bicubic	Lanczos	SRCNN	SRGAN
Poor (1)	0	0	0	0	0	0
Reasonable (2)	1	1	1	1	1	0
Good (3)	2	2	2	2	2	3
Very high (4)	2	2	2	2	2	2
**Experts (all)**						
Visual quality perception (MOS score)	Number of cases					
	Nearest	Bilinear	Bicubic	Lanczos	SRCNN	SRGAN
Poor (1)	9	11	7	2	2	1
Reasonable (2)	6	6	12	13	10	4
Good (3)	8	6	4	7	9	11
Very high (4)	2	2	2	3	4	9
**Lay participants (all)**						
Visual quality perception (MOS score)	Number of cases					
	Nearest	Bilinear	Bicubic	Lanczos	SRCNN	SRGAN
Poor (1)	41	16	8	5	2	2
Reasonable (2)	30	45	39	27	23	24
Good (3)	11	20	32	46	49	36
Very high (4)	3	4	6	7	11	23
**All observers**						
Visual quality perception (MOS score)	Number of cases					
	Nearest	Bilinear	Bicubic	Lanczos	SRCNN	SRGAN
Poor (1)	50	27	15	7	4	3
Reasonable (2)	36	51	51	40	33	28
Good (3)	19	26	36	53	58	47
Very high (4)	5	6	8	10	15	32

**Table 2:** Values of area under the curve (AUC) achieved for each M_A_–M_B_ considering all participants, and the expert and lay participant groups.

M_A_–M_B_ Pair	Experts (All)	Lay (All)	All Observers
Nearest–Bilinear	0.454	0.652	0.544
Nearest–Bicubic	0.479	0.733	0.602
Nearest–Lanczos	0.592	0.791	0.692
Nearest–SRCNN	0.634	0.829	0.731
Nearest–SRGAN	0.764	0.839	0.797
Bilinear–Bicubic	0.535	0.600	0.559
Bilinear–Lanczos	0.648	0.682	0.657
Bilinear–SRCNN	0.683	0.733	0.701
Bilinear–SRGAN	0.796	0.748	0.775
Bicubic–Lanczos	0.632	0.586	0.605
Bicubic–SRCNN	0.675	0.641	0.656
Bicubic–SRGAN	0.804	0.667	0.741
Lanczos–SRCNN	0.556	0.557	0.557
Lanczos–SRGAN	0.720	0.596	0.662
SRCNN–SRGAN	0.668	0.545	0.610

**Table 3:** Average and standard deviation of mean square error (MSE), peak signal-to-noise ratio (PSNR), and structural similarity index (SSIM) computed for each method.

Methods	MSE	PSNR	SSIM
SRGAN	3.521 (±0.931)	42.771 (±1.038)	1.000 (±0.000)
SRCNN	16.606 (±3.035)	35.984 (±0.766)	0.983 (±0.001)
Lanczos	15.416 (±2.732)	36.303 (±0.739)	0.974 (±0.003)
Bicubic	17.862 (±3.145)	35.663 (±0.736)	0.974 (±0.003)
Bilinear	21.371 (±3.517)	34.878 (±0.695)	0.970 (±0.003)
Nearest	40.622 (±5.805)	32.078 (±0.616)	0.953 (±0.005)

**Table 4:** Confusion matrix results for each ResNet model.

**ResNetNearest**	*n* = 104		Predicted	
			Healthy	PBL
	Actual	Healthy	19	33
		PBL	3	49
**ResNetBilinear**	*n* = 104		Predicted	
			Healthy	PBL
	Actual	Healthy	26	26
		PBL	2	50
**ResNetBicubic**	*n* = 104		Predicted	
			Healthy	PBL
	Actual	Healthy	38	14
		PBL	13	39
**ResNetLanczos**	*n* = 104		Predicted	
			Healthy	PBL
	Actual	Healthy	49	3
		PBL	27	25
**ResNetSRCNN**	*n* = 104		Predicted	
			Healthy	PBL
	Actual	Healthy	33	19
		PBL	5	47
**ResNetSRGAN**	*n* = 104		Predicted	
			Healthy	PBL
	Actual	Healthy	46	6
		PBL	21	31

**Table 5:** Confusion matrix results for each Inception model.

**Inception_Nearest_**	*n* = 104		Predicted	
			Healthy	PBL
	Actual	Healthy	32	20
		PBL	2	50
**Inception_Bilinear_**	*n* = 104		Predicted	
			Healthy	PBL
	Actual	Healthy	25	27
		PBL	5	74
**Inception_Bicubic_**	*n* = 104		Predicted	
			Healthy	PBL
	Actual	Healthy	37	15
		PBL	4	48
**Inception_Lanczos_**	*n* = 104		Predicted	
			Healthy	PBL
	Actual	Healthy	25	27
		PBL	1	51
**Inception_SRCNN_**	*n* = 104		Predicted	
			Healthy	PBL
	Actual	Healthy	32	20
		PBL	9	43
**Inception_SRGAN_**	*n* = 104		Predicted	
			Healthy	PBL
	Actual	Healthy	36	16
		PBL	10	42

**Table 6:** Test results for each ResNet model.

Metric	ResNet_Nearest_	ResNet_Bilinear_	ResNet_Bicubic_	ResNet_Lanczos_	ResNet_SRCNN_	ResNet_SRGAN_
PPV (precision)	0.598	0.658	0.736	0.893	0.712	0.838
Sensitivity (recall)	0.942	0.962	0.750	0.481	0.904	0.596
Specificity	0.365	0.500	0.731	0.942	0.635	0.885
NPV	0.864	0.929	0.745	0.645	0.868	0.687
AUC-ROC curve	0.749	0.811	0.864	0.833	0.877	0.822
AUC-PR curve	0.712	0.769	0.868	0.836	0.886	0.807

**Table 7:** Test results for each Inception model.

Metric	Inception_Nearest_	Inception_Bilinear_	Inception_Bicubic_	Inception_Lanczos_	Inception_SRCNN_	Inception_SRGAN_
PPV (precision)	0.714	0.635	0.762	0.654	0.683	0.724
Sensitivity (recall)	0.962	0.904	0.923	0.981	0.827	0.808
Specificity	0.615	0.481	0.711	0.481	0.615	0.692
NPV	0.941	0.833	0.902	0.962	0.780	0.783
AUC-ROC curve	0.811	0.756	0.860	0.873	0.817	0.824
AUC-PR curve	0.824	0.718	0.847	0.867	0.818	0.806

**Table 8:** Overall test accuracy obtained for each model.

Method	Accuracy
ResNet_Nearest_	0.654
ResNet_Bilinear_	0.731
ResNet_Bicubic_	0.740
ResNet_Lanczos_	0.712
ResNet_SRCNN_	0.769
ResNet_SRGAN_	0.740
Inception_Nearest_	0.788
Inception_Bilinear_	0.952
Inception_Bicubic_	0.817
Inception_Lanczos_	0.731
Inception_SRCNN_	0.721
Inception_SRGAN_	0.750

## Data Availability

The data presented in this study are available on request from the corresponding author. The data are not publicly available due to privacy and ethical restrictions.

## References

[1] Jeffcoat M.K., Wang I.C., Reddy M.S. (1995). Radiographic Diagnosis in Periodontics. Periodontol. 2000.

[2] Tugnait A., Clerehugh V., Hirschmann P.N. (2000). The Usefulness of Radiographs in Diagnosis and Management of Periodontal Diseases: A Review. J. Dent..

[3] Estrela C., Bueno M.R., Leles C.R., Azevedo B., Azevedo J.R. (2008). Accuracy of Cone Beam Computed Tomography and Panoramic and Periapical Radiography for Detection of Apical Periodontitis. J. Endod..

[4] Tugnait A., Clerehugh V., Hirschmann P.N. (2000). Survey of Radiographic Practices for Periodontal Disease in UK and Irish Dental Teaching Hospitals. Dentomaxillofac Radiol.

[5] Douglass C.W., Valachovic R.W., Wijesinha A., Chauncey H.H., Kapur K.K., McNeil B.J. (1986). Clinical Efficacy of Dental Radiography in the Detection of Dental Caries and Periodontal Diseases. Oral Surg. Oral Med. Oral Pathol..

[6] Pepelassi E.A., Diamanti-Kipioti A. (1997). Selection of the Most Accurate Method of Conventional Radiography for the Assessment of Periodontal Osseous Destruction. J. Clin. Periodontol.

[7] Rohlin M., Akesson L., Hakansson J., Hakansson H., Nasstrom K. (1989). Comparison between Panoramic and Periapical Radiography in the Diagnosis of Periodontal Bone Loss. Dentomaxillofacial Radiol..

[8] Krois J., Ekert T., Meinhold L., Golla T., Kharbot B., Wittemeier A., Schwendicke F. (2019). Deep Learning for the Radiographic Detection of Periodontal Bone Loss. Sci. Rep..

[9] Faria M.D.B. (1997). Quantitative Analysis of Radiation Dose for Critical Organs during Linear Tomography Regarding Intraosseous Dental Implant Planning. Master’s Thesis.

[10] Baskan O., Erol C., Ozbek H., Paksoy Y. (2015). Effect of Radiation Dose Reduction on Image Quality in Adult Head CT with Noise-Suppressing Reconstruction System with a 256 Slice MDCT. J. Appl. Clin. Med Phys..

[11] De Morais J., Sakakura C., Loffredo L., Scaf G. (2006). Accuracy of Zoomed Digital Image in the Detection of Periodontal Bone Defect: In Vitro Study. Dentomaxillofacial Radiol..

[12] Kositbowornchai S., Basiw M., Promwang Y., Moragorn H., Sooksuntisakoonchai N. (2004). Accuracy of Diagnosing Occlusal Caries Using Enhanced Digital Images. Dentomaxillofacial Radiol..

[13] Alvares H. (2019). D Analysis of the Impact of Image Interpolation Methods in the Segmentation of Skin Lesions Using the SegNet Convolutional Neural Network.

[14] Goodfellow I., Bengio Y., Courville A. (2016). Deep Learning (Adaptive Computation and Machine Learning).

[15] Dodge S., Karam L. Understanding How Image Quality Affects Deep Neural Networks. Proceedings of the 8th International Conference on Quality of Multimedia Experience (QoMEX).

[16] Koziarski M., Cyganek B. (2018). Impact of Low Resolution on Image Recognition with Deep Neural Networks: An Experimental Study. Int. J. Appl. Math. Comput. Sci..

[17] Chatfield K., Simonyan K., Vedaldi A., Zisserman A. Return of the Devil in the Details: Delving Deep into Convolutional Nets. Proceedings of the British Machine Vision Conference 2014.

[18] Szegedy C., Wei L., Yangqing J., Sermanet P., Reed S., Anguelov D., Erhan D., Vanhoucke V., Rabinovich A. Going Deeper with Convolutions. Proceedings of the 2015 IEEE Conference on Computer Vision and Pattern Recognition (CVPR).

[19] Simonyan K., Zisserman A. (2014). Very Deep Convolutional Networks for Large-Scale Image Recognition. arXiv.

[20] Krizhevsky A., Sutskever I., Hinton G.E. (2017). ImageNet Classification with Deep Convolutional Neural Networks. Commun. Acm..

[21] Moran M.B.H., Faria M.D.B., Giraldi G.A., Bastos L.F., Conci A. (2021). Using Super-Resolution Generative Adversarial Network Models and Transfer Learning to Obtain High Resolution Digital Periapical Radiographs. Comput. Biol. Med..

[22] Zeng K., Zheng H., Cai C., Yang Y., Zhang K., Chen Z. (2018). Simultaneous Single- and Multi-Contrast Super-Resolution for Brain MRI Images Based on a Convolutional Neural Network. Comput. Biol. Med..

[23] Zhang Y., An M. (2017). Deep Learning- and Transfer Learning-Based Super Resolution Reconstruction from Single Medical Image. J. Healthc. Eng..

[24] Hatvani J., Basarab A., Tourneret J.-Y., Gyongy M., Kouame D. (2019). A Tensor Factorization Method for 3-D Super Resolution with Application to Dental CT. IEEE Trans. Med. Imaging.

[25] Umehara K., Ota J., Ishida T. (2018). Application of Super-Resolution Convolutional Neural Network for Enhancing Image Resolution in Chest CT. J. Digit. Imaging.

[26] Park J., Hwang D., Kim K.Y., Kang S.K., Kim Y.K., Lee J.S. (2018). Computed Tomography Super-Resolution Using Deep Convolutional Neural Network. Phys. Med. Biol..

[27] Båth M., Zachrisson S., Månsson L.G. VGC Analysis: Application of the ROC Methodology to Visual Grading Tasks. Proceedings of the Medical Imaging 2008: Image Perception, Observer Performance, and Technology Assessment.

[28] Perschbacher S. (2014). Periodontal Diseases. Oral Radiology: Principles and Interpretation.

[29] Moran M.B.H., Faria M.D.B., Giraldi G.A., Bastos L.F., Inacio B., Conci A. On Using Convolutional Neural Networks to Classify Periodontal Bone Destruction in Periapical Radiographs. Proceedings of the 2020 IEEE International Conference on Bioinformatics and Biomedicine (BIBM).

[30] Lin P.L., Huang P.Y., Huang P.W. (2017). Automatic Methods for Alveolar Bone Loss Degree Measurement in Periodontitis Periapical Radiographs. Comput. Methods Programs Biomed..

[31] Lee J.-H., Kim D., Jeong S.-N., Choi S.-H. (2018). Diagnosis and Prediction of Periodontally Compromised Teeth Using a Deep Learning-Based Convolutional Neural Network Algorithm. J. Periodontal Implant. Sci..

[32] Carmody D.P., McGrath S.P., Dunn S.M., van der Stelt P.F., Schouten E. (2001). Machine Classification of Dental Images with Visual Search. Acad. Radiol..

[33] Mol A., van der Stelt P.F. (1992). Application of Computer-Aided Image Interpretation to the Diagnosis of Periapical Bone Lesions. Dentomaxillofacial Radiol..

[34] Ekert T., Krois J., Meinhold L., Elhennawy K., Emara R., Golla T., Schwendicke F. (2019). Deep Learning for the Radiographic Detection of Apical Lesions. J. Endod..

[35] Yang C.-Y., Ma C., Yang M.-H., Fleet D., Pajdla T., Schiele B., Tuytelaars T. (2014). Single-Image Super-Resolution: A Benchmark. Proceedings of the Computer Vision–ECCV 2014.

[36] Shi J., Liu Q., Wang C., Zhang Q., Ying S., Xu H. (2018). Super-Resolution Reconstruction of MR Image with a Novel Residual Learning Network Algorithm. Phys. Med. Biol..

[37] Zhao C., Shao M., Carass A., Li H., Dewey B.E., Ellingsen L.M., Woo J., Guttman M.A., Blitz A.M., Stone M. (2019). Applications of a Deep Learning Method for Anti-Aliasing and Super-Resolution in MRI. Magn. Reson. Imaging.

[38] Dong C., Loy C.C., He K., Tang X. (2016). Image Super-Resolution Using Deep Convolutional Networks. IEEE Trans. Pattern Anal. Mach. Intell..

[39] Ledig C., Theis L., Huszar F., Caballero J., Cunningham A., Acosta A., Aitken A., Tejani A., Totz J., Wang Z. Photo-Realistic Single Image Super-Resolution Using a Generative Adversarial Network. Proceedings of the 2017 IEEE Conference on Computer Vision and Pattern Recognition (CVPR).

[40] Martin D., Fowlkes C., Tal D., Malik J. A Database of Human Segmented Natural Images and Its Application to Evaluating Segmentation Algorithms and Measuring Ecological Statistics. Proceedings of the Proceedings Eighth IEEE International Conference on Computer Vision (ICCV 2001).

[41] Qiu D., Zhang S., Liu Y., Zhu J., Zheng L. (2020). Super-Resolution Reconstruction of Knee Magnetic Resonance Imaging Based on Deep Learning. Comput. Methods Programs Biomed..

[42] Blau Y., Mechrez R., Timofte R., Michaeli T., Zelnik-Manor L., Leal-Taixé L., Roth S. (2019). The 2018 PIRM Challenge on Perceptual Image Super-Resolution. Computer Vision–ECCV 2018 Workshops.

[43] Nagano Y., Kikuta Y. SRGAN for Super-Resolving Low-Resolution Food Images. Proceedings of the Joint Workshop on Multimedia for Cooking and Eating Activities and Multimedia Assisted Dietary Management.

[44] Xiong Y., Guo S., Chen J., Deng X., Sun L., Zheng X., Xu W. (2020). Improved SRGAN for Remote Sensing Image Super-Resolution Across Locations and Sensors. Remote Sens..

[45] Liu J., Chen F., Wang X., Liao H., Zhu D., Yan J., Huang H., Shen L., Thompson P.M., Westin C.-F., Pennec X., Joshi S., Nielsen M., Fletcher T. (2019). An Edge Enhanced SRGAN for MRI Super Resolution in Slice-Selection Direction. Multimodal Brain Image Analysis and Mathematical Foundations of Computational Anatomy.

[46] (2010). Kwang In Kim; Younghee Kwon Single-Image Super-Resolution Using Sparse Regression and Natural Image Prior. IEEE Trans. Pattern Anal. Mach. Intell..

[47] Jianchao Y., Wright J., Huang T.S. (2010). Yi Ma Image Super-Resolution Via Sparse Representation. IEEE Trans. Image Process..

[48] Timofte R., De Smet V., Van Gool L. A+: Adjusted Anchored Neighborhood Regression for Fast Super-Resolution. Proceedings of 12th Asian Conference on Computer Vision (ACCV 2014).

[49] Seitzer M., Yang G., Schlemper J., Oktay O., Würfl T., Christlein V., Wong T., Mohiaddin R., Firmin D., Keegan J., Frangi A.F., Schnabel J.A., Davatzikos C., Alberola-López C., Fichtinger G. (2018). Adversarial and Perceptual Refinement for Compressed Sensing MRI Reconstruction. Medical Image Computing and Computer Assisted Intervention – MICCAI 2018.

[50] He K., Zhang X., Ren S., Sun J. Deep Residual Learning for Image Recognition. Proceedings of the 2016 IEEE Conference on Computer Vision and Pattern Recognition (CVPR).

[51] Leung H., Haykin S. (1991). The Complex Backpropagation Algorithm. IEEE Trans. Signal. Process..

[52] Russakovsky O., Deng J., Su H., Krause J., Satheesh S., Ma S., Huang Z., Karpathy A., Khosla A., Bernstein M. (2015). ImageNet Large Scale Visual Recognition Challenge. IntJ. Comput. Vis..

[53] Ramsey P.H., Hodges J.L., Popper Shaffer J. (1993). Significance Probabilities of the Wilcoxon Signed-Rank Test. J. Nonparametric Stat..

[54] Powers D. (2007). Evaluation-From Precision, Recall and F-Measure to ROC. J. Mach. Lear Tech..

[55] Pepe A., Li J., Rolf-Pissarczyk M., Gsaxner C., Chen X., Holzapfel G.A., Egger J. (2020). Detection, Segmentation, Simulation and Visualization of Aortic Dissections: A Review. Med. Image Anal..

